# Correlates of Social Cognition and Psychopathic Traits in a Community-Based Sample of Males

**DOI:** 10.3389/fpsyg.2021.656299

**Published:** 2021-04-30

**Authors:** Grace A. Carroll, V. Tamara Montrose, Tom Burke

**Affiliations:** ^1^School of Psychology, Queens University Belfast, Belfast, United Kingdom; ^2^Independent Researcher, Manchester, United Kingdom; ^3^School of Psychology, University College Dublin, Dublin, Ireland; ^4^School of Psychology, National University of Ireland Galway, Galway, Ireland

**Keywords:** social cognition, psychopathic traits, psychometrics, short forms, Reading the Mind in the Eyes Test

## Abstract

Social cognition is the ability to identify, understand, and interpret mental states and emotions. Psychopathic traits are typically described in two ways; Primary: shallow affect, emotional detachment, and relationship difficulties, and Secondary Psychopathic Traits: antisocial traits, impulsiveness, and emotional dysregulation. People with high psychopathic traits tend to perform lower on measures of social cognition. This study investigated the relationship of social cognition (mentalising) to primary and secondary psychopathic traits in a non-clinical sample, and investigated the psychometric properties of the Reading the Mind in the Eyes Test (RMET) Short Forms (A and B). A community-based male sample (*N* = 1,000; age range 18–78) was recruited through an online platform. Psychopathic traits were measured using Levenson, Kiehl, and Fitzpatrick's Self-Report Psychopathy Scale, and stratified into Primary and Secondary Psychopathic traits. Secondary validation of the RMET Short Forms was completed investigating scale reliability, and validity. Findings suggest excellent psychometrics in a large community cohort for the RMET Short Forms (A and B), with significant negative correlations on social cognitive performance and high self-report psychopathy. The item valence within the social cognitive measure (positive, negative, and neutral affect stimuli) was also examined, and correlated significantly with both Primary and Secondary Psychopathic traits. This study provides further validation of the RMET Short Forms (A and B), and adds to the literature on the scale by investigating performance on short-form specific valence. This study further suggests that in a non-clinical community sample of males, that higher psychopathic traits correlated significantly, and negatively, with social cognitive performance.

## Introduction

Social Cognition is a broad concept which incorporates understanding the intentions, beliefs, emotions, and mental states of others i.e., affective theory of mind, while also considering social interaction, social context, and social decision-making i.e., cognitive theory of mind (Alcalá-López et al., 2019). Social cognition is a distinct cognitive concept much like memory, language, and attention with individual differences in a persons' potential ability level and tendency to use these skills. Attentional dysfunction and deficits in understanding the emotional cues of others may partially explain aggression and anti-social behaviour seen in humans, and certain personality traits are also known to negative correlate with social cognition (Edalati et al., 2016). This may be due to a reduced capacity for emotional empathy, even though cognitive empathy may be intact e.g., little remorse or feeling for a negative action, even though it is known to be the wrong thing to do, or not socially acceptable. There have been suggestions within the literature that a deficiency in the ability to understand other people's mental states may be related to antisocial behaviour, aggressive behaviour, and/or psychopathy (Richell et al., 2003). People with higher rates of psychopathic traits are reported to show less empathy, and reduced preference towards both humans and animals compared to individuals low in psychopathic traits (Carroll et al., 2020). Psychopathy is characterised by callousness, manipulative behaviour, superficial charm, shallow affects, irresponsibility, lack of remorse, and antisocial behaviour (Hare et al., 1991). Psychopathy is a multidimensional construct, with several proposed factor structures. However, the two-factor conceptualisation, i.e., Primary and Secondary psychopathy, remains the most widely accepted within the literature (see Sellbom and Drislane, 2020 for more). *Primary psychopathy* has been thought to constitute the core features of psychopathy, including emotional detachment, shallow affect, and failure to form deep relationships while *Secondary psychopathy* is strongly related to antisocial lifestyle, impulsiveness, and emotional disturbance (Takamatsu and Takai, 2019).

There have been mixed findings within the literature regarding the association between psychopathy and measures of social cognition. Some researchers propose that psychopathy is associated with deficits in the ability to recognise and interpret the emotional state of others (e.g., Blair, 2006, 2019; Blair et al., 2006; Blair and Zhang, 2020). However, others argue that individuals with psychopathic traits have a similar, or even enhanced ability to perceive the emotions of others, in comparison to the general population (e.g., Wheeler et al., 2009; Book et al., 2013). It has also been suggested an enhanced ability to take the perspective of others may allow individuals with elevated psychopathic traits to detect and subsequently manipulate vulnerable individuals. The *Reading the Mind in the Eyes Test* (RMET; Baron-Cohen et al., 2001) is a measure of affective ToM and has been used to assess associations between psychopathy and the social cognitive process referred to as affective theory of mind or mentalising (Ali and Chamorro-Premuzic, 2010). While some authors have found a negative association between psychopathic traits and RMET scores (e.g., Ali and Chamorro-Premuzic, 2010; Sandvik et al., 2014), others have found no such association (e.g., Richell et al., 2003).

These mixed findings may be due to variation in the population under examination (e.g., forensic, student or community sample), the gender breakdown of the participants (e.g., all male, all female, mostly female), the type of test administered (self-report or clinical assessment), and the way in which psychopathy was conceptualised (e.g., single construct, two-factor or three-factor). In addition, the sample size used was often relatively small. For example, Ali and Chamorro-Premuzic (2010) sampled 112 undergraduates, 92 of which were female. As such, more research is needed using a larger sample size to consider the relationship between social cognition and psychopathic traits within a homogeneous community sample. Consequently, the aim of this study was to assess associations between self-report psychopathy and RMET scores with a large community sample of males. Male participants were recruited because men are more likely to have social cognition disorders and typically display higher levels of psychopathy than females (Zilioli et al., 2015; Lobbestael et al., 2020). A second aim was to investigate the relationship between recently developed RMET Short Forms A and B (Burke et al., 2020), and a well-validated measure of primary and secondary psychopathic traits (Levenson et al., 1995), as well as cross-validate the short forms of the Reading the Mind in the Eyes Test to the full measure. Based on the literature in relation to social cognition and psychopathy in a community sample, we hypothesise that participants with higher levels of self-reported psychopathy will perform lower on the RMET. We further hypothesise that this pattern of scoring should be similar across the short-forms of the RMET, inclusive of the direction of the association.

## Materials and Methods

### Participants and Procedure

Data from *N* = 1,000 participants were gathered using Prolific Academic©, an online platform for survey-based data collection. In terms of eligibility criteria, participants were required to be over the age of 18, to give explicit consent for data usage, and male. Exclusion criteria included not being male; having neurological or mental health diagnoses which may interfere with test performance; and being non-native English speakers.

Participants were screened for exclusion criteria prior to engaging with the study. Participants who expressed interest in taking part clicked a link which brought them to an information sheet detailing the study. Following this, they provided consent. Participants provided demographic details, and then proceeded to complete the measures. On completion of the survey, participants received a gratuity equivalent to an average of £5.00 per hour. A pilot study was conducted with *n* = 10 participants, with no changes made following this. Consequently, the study was continued and the data from these *n* = 10 was retained.

### Measures

Participants provided basic demographic information such as age, sex, student status, residence, and employment status. Demographics, social cognition (mentalising), and psychopathy outcomes were gathered using the online platform, Prolific©. This platform has been shown to have high data quality, a diverse participant pool, and demonstrates reproducibility of known effects (Peer et al., 2017; Palan and Schitter, 2018).

The Reading the Mind in the Eyes Task (RMET-36) (Baron-Cohen et al., 2001) is a 36-item assessment where black and white photographs of eye regions are presented, and participants are required to infer mental/emotional states from four choices e.g., terrified, upset, bored, irritated. The RMET-36 can also provide individual scores for Positive, Negative, and Neutral valence (Hudson et al., 2020). Examples of Positive valence include: Friendly (Q20), Reflective (Q29), and Flirtatious (Q30); Negative valence includes: Hostile (Q26), Uneasy (Q7), and Upset (Q2); Neutral valence include Preoccupied (Q22) and Pensive (Q24), among others outlined by Hudson et al. (2020). Recently, two Short Forms (A and B) were developed (Burke et al., 2020) which were considered within this study. The RMET Short Forms (A and B) are 18-item versions of the full-scale and have been shown to have equivalency and excellent psychometric properties. The ordered administration of the RMET-36 and RMET Short forms (A and B) is consistent in this study with the original study outlining their development (Burke et al., 2020). The RMET has further been validated using remote administration via survey-based platforms (Khorashad et al., 2015).

The Levenson Self-Report Psychopathy Scale (LSRPS) is a 26-item self-report measure designed to evaluate both the behavioural and personality traits commonly associated with psychopathy in the literature (Levenson et al., 1995). Each item consists of a statement that the participant reads and then endorses on a 5-point Likert scale (disagree strongly to agree strongly). Some items are reverse scored to control for response sets. The measure has two distinct factors (1) Primary Psychopathy (a measure of callous or manipulative interpersonal style); and (2) Secondary Psychopathy (a measure of poor behavioural control, and failure to learn from mistakes).

### Ethical Considerations

The Queens University Belfast Research Ethics Committee approved this study (REC REF: EPS 20_32). All procedures were conducted in accordance with the principles expressed in the Declaration of Helsinki. Each participant provided consent to take part in the study prior to completion of the measures.

### Statistical Analysis

Demographic characteristics and outcome data are reported as means, standard deviations, and frequencies. Classification for good internal consistency, using Cronbach's alpha, remains at the internationally accepted value >0.70. ANOVA were employed for significance testing e.g., comparing outcomes following stratification into Upper (*n* = 250) and Lower (*n* = 250) groups. The difficulty and discrimination coefficient for each of the RMET Short Forms was computed to investigate consistency with the original validation (Burke et al., 2020). A detailed account of the discrimination coefficient and difficulty coefficient can be found within the original validation study (Burke et al., 2016, 2020). Correlation analyses were carried out to examine associations between self-reported psychopathic traits and social cognition using the RMET-36, RMET-A and RMET-B. Participants were stratified into Upper and Lower quartiles based on their RMET-36 item performance and following this significance testing and correlations were conducted.

## Results

Participants in this study had a wide age range (18–78 years) with an average of 37.44 ± 13.99 as reported in Table 1 alongside the outcome data. The majority of participants were engaged in gainful employment (64.8%) or a student (19.4%).

**Table 1:** Participant demographics and outcome data, stratified by upper and lower quartile performance on the RMET.

**Variable**		**Total (*N* = 1,000)**	**Upper (*N* = 250)**	**Lower (*N* = 250)**
Age [years (range)]		37.44 ± 13.99 [18–78]	38.59 ± 14.43 [18–75]	34.83 ± 12.77 [18–88]
Employment	Full-time	53.7%	49.6%	51.6%
	Part-time	11.1%	11.6%	11.6%
	Not gainful	10.2%	9.2%	9.6%
	Unemployed	11.7%	14.4%	10.4%
	Other	7.5%	11.6%	5.6%
Student status (yes)		19.4%	18.4%	22.8%
RMET-36		24.91 ± 5.20	30.54 ± 1.41	17.78 ± 3.83
RMET-36 Valence	Positive % correct	71.02 ± 16.52	84.92 ± 8.21	51.92 ± 15.94
	Negative % correct	65.14 ± 18.03	82.00 ± 10.10	46.12 ± 15.15
	Neutral % correct	72.56 ± 21.87	89.82 ± 11.63	49.60 ± 21.00
RMET—A		12.67 ± 2.92	15.50 ± 1.23	9.08 ± 2.36
RMET—B		12.24 ± 2.88	15.03 ± 1.28	8.70 ± 2.35
LSRPS—Primary		2.25 ± 0.632	2.04 ± 0.601	2.50 ± 0.656
LSRPS—Secondary		2.41 ± 0.560	2.33 ± 0.52	2.50 ± 0.591

*All participants in this study are Male*.

### RMET Short Form Psychometrics

Good internal consistency was found for the RMET scales (>0.75). For the total cohort, there were significant positive associations between the RMET-36 and the RMET-A (*r* = 0.898; *p* < 0.001), as well as the RMET-36 and the RMET-B (*r* = 0.895; *p* < 0.001). The RMET-A and RMET-B were also significantly associated (*r* = 0.608; *p* < 0.001). In terms of the difficulty coefficient, the RMET-A and RMET-B both have consistent “Medium Difficulty” coefficients (A: 68.29%; B: 65.91%) compared to the original validation study (Burke et al., 2020; A: 68.4%; B: 69.6%). When compared using ANOVA, there were no significant differences between the total group's performance on the RMET-A compared to the RMET-B. There were no significant difference between the Upper quartile's performance between the RMET-A and RMET-B. There was also no significant difference between the Lower quartile's performance when comparing the RMET-A and RMET-B. Comparing performance stratified by Upper and Lower quartiles on both short forms yielded significant differences (*p* < 0.001).

### Social Cognition and Psychopathy Correlates

**Figure 1:**
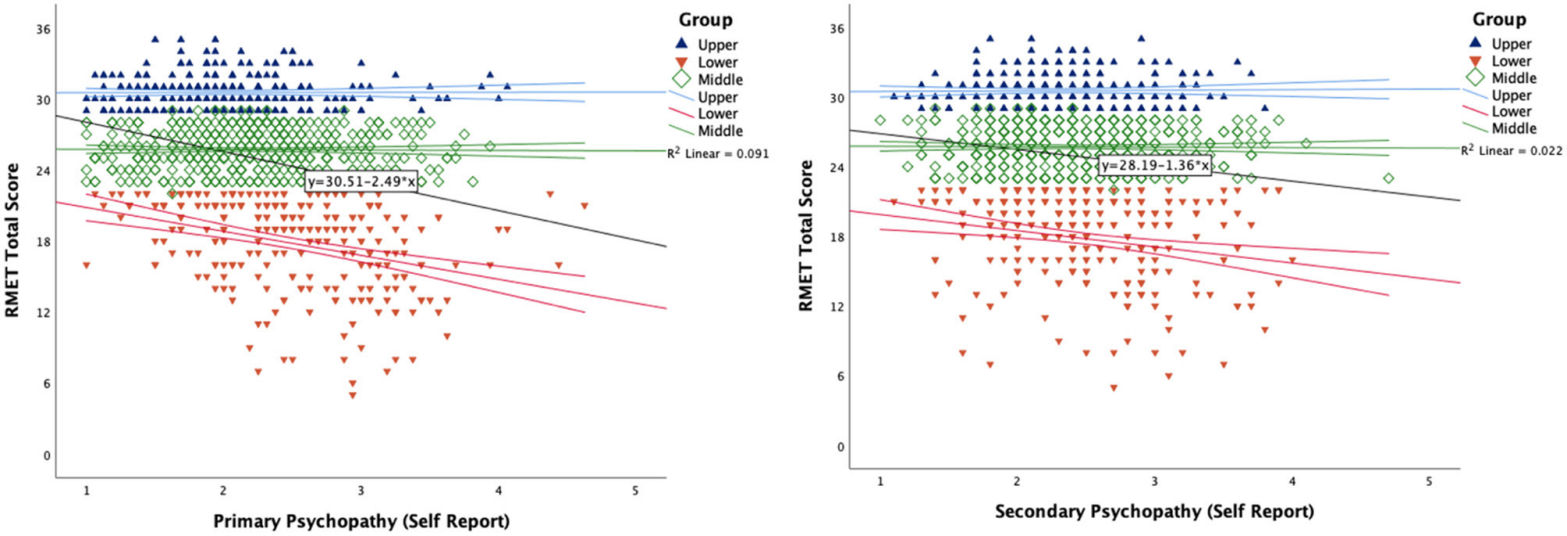
Correlation outcome data on the RMET-36 as well as self-reported outcome data from the Primary (Left) and Secondary (Right). Both figures illustrate the total group, with colour-figure demarcation of those stratified within the Upper Quartile (*n* = 250), the Lower Quartile (*n* = 250), and those in the Middle group (*n* = 500). These figures illustrate the negative relationship from the Lower Quartile between their performance on the RMET total and self-report psychopathy scores.

For the total cohort, there was a negative association between the RMET-36 and primary psychopathic traits (*r* = −0.302; *p* < 0.001), as well as secondary psychopathic trait scores (*r* = −0.148; *p* < 0.001), as seen in Figure 1. Performance on the Positive, Negative, and Neutral RMET-36 was also negatively and significantly associated with primary and secondary psychopathic traits, as reported in Table 2. The RMET-A and RMET-B were also associated with the primary psychopathic scores (A: *r* = −0.269; *p* < 0.001; B: *r* = −0.273; *p* < 0.001) and secondary psychopathic scores (A: *r* = −0.097; *p* = 0.002; B: *r* = −0.169; *p* < 0.001).

**Table 2:** Correlation coefficients for the total group (*N* = 1,000).

		**RMET Total**	**RMET A**	**RMET B**	**Primary psychopathy**	**Secondary psychopathy**	**Valence: Positive**	**Valence: Neutral**	**Valence: Negative**
RMET total	*r*	1							
	*p*-value	–							
RMET A	*r*	0.898^**^	1						
	*p*-value	≤0.001	-						
RMET B	*r*	0.895^**^	0.608^**^	1					
	*p*-value	≤0.001	≤0.001	-					
Primary psychopathy	*r*	−0.302^**^	−0.269^**^	−0.273^**^	1				
	*p*-value	≤0.001	≤0.001	≤0.001	-				
Secondary psychopathy	*r*	−0.148^**^	−0.097^**^	−0.169^**^	0.408^**^	1			
	*p*-value	≤0.001	0.002	≤0.001	≤0.001	-			
Valence: Positive	*r*	0.839^**^	0.738^**^	0.767^**^	−0.247^**^	−0.161^**^	1		
	*p*-value	≤0.001	≤0.001	≤0.001	≤0.001	≤0.001	-		
Valence: Neutral	*r*	0.758^**^	0.630^**^	0.730^**^	−0.253^**^	−0.108^**^	0.522^**^	1	
	*p*-value	≤0.001	≤0.001	≤0.001	≤0.001	≤0.001	≤0.001	-	
Valence: Negative	*r*	0.780^**^	0.752^**^	0.646^**^	−0.228^**^	−0.076^*^	0.394^**^	0.441^**^	1
	*p*-value	≤0.001	≤0.001	≤0.001	≤0.001	0.018	≤0.001	≤0.001	-

* *Correlation is significant at the 0.05 level (2-tailed)*:

** *Correlation is significant at the 0.01 level (2-tailed). Valence refers to total % correct of positive, neutral, and negative valence, respectively. RMET, Reading the Mind in the Eyes Test*.

With regards to the psychopathic traits, considering participants who performed in the Upper quartile on the RMET-36 first, the only significant associations were between the primary and secondary subscales (*r* = 0.368; *p* < 0.001). By way of contrast, in the lower quartile, the primary psychopathic scale was negatively associated with the RMET-36 (*r* = −0.346; *p* < 0.001), RMET-A (*r* = −0.271; *p* < 0.001), and the RMET-B (*r* = −0.291; *p* < 0.001). A similar association pattern was observed for secondary psychopathic traits and the RMET-36 (*r* = −0.215; *p* < 0.001), RMET-A (*r* = −0.142; *p* = 0.025), and the RMET-B (*r* = −0.207; *p* = 0.001) as seen in Table 3. Figure 1 illustrates these relationship and trends for the stratified cohorts together, which considers the total cohort. What can be observed is the association between the RMET lower quartile cohort (*n* = 250) and their psychopathy outcomes, relative to the upper (*n* = 250), and the middle (*n* = 500) cohorts.

**Table 3:** Correlation coefficients for the Upper quartile (*n* = 250) and Lower quartile (*n* = 250).

**Variable**		**RMET total**	**RMET A**	**RMET B**	**Primary psychopathy**	**Secondary psychopathy**	**Valence: Positive**	**Valence: Neutral**	**Valence: Negative**
RMET total	*r*	1	0.539^**^	0.581^**^	0.004	0.020	0.428^**^	0.280^**^	0.474^**^
	*p-value*	-	≤0.001	≤0.001	0.945	0.754	≤0.001	≤0.001	≤0.001
RMET A	*r*	0.813^**^	1	0.371^**^	0.039	0.051	0.140^*^	0.086	0.387^**^
	*p-value*	≤0.001	-	≤0.001	0.534	0.423	0.027	0.173	≤0.001
RMET B	*r*	0.811^**^	0.318^**^	1	−0.033	−0.027	0.336^**^	0.226^**^	0.149^*^
	*p-value*	≤0.001	≤0.001	-	0.600	0.668	≤0.001	≤0.001	0.018
Primary psychopathy	*r*	−0.346^**^	−0.271^**^	−0.291^**^	1	0.368^**^	−0.034	−0.034	0.060
	*p-value*	≤0.001	≤0.001	≤0.001	-	≤0.001	0.592	0.598	0.348
Secondary psychopathy	*r*	−0.215^**^	−0.142^*^	−0.207^**^	0.505^**^	1	−0.004	0.064	−0.014
	*p-value*	≤0.001	0.025	≤0.001	≤0.001	-	0.950	0.317	0.827
Valence: Positive	*r*	0.749^**^	0.568^**^	0.649^**^	−0.226^**^	−0.235^**^	1	−0.200^**^	−0.416^**^
	*p-value*	≤0.001	≤0.001	≤0.001	≤0.001	≤0.001	-	0.001	≤0.001
Valence: Neutral	*r*	0.603^**^	0.397^**^	0.584^**^	−0.245^**^	−0.110	0.263^**^	1	−0.118
	*p-value*	≤0.001	≤0.001	≤0.001	≤0.001	0.083	≤0.001	-	0.062
Valence: Negative	*r*	0.526^**^	0.550^**^	0.303^**^	−0.198^**^	−0.032	−0.032	0.088	1
	*p-value*	≤0.001	≤0.001	≤0.001	0.002	0.615	0.609	0.166	-

* *Correlation is significant at the 0.05 level (2-tailed)*;

** *Correlation is significant at the 0.01 level (2-tailed). **Upper Quartile** Cohort represented above the dashed line; **Lower Quartile** Cohort represented below the dashed line*.

## Discussion

An aspect of affective social cognition (mentalising) was considered for this study, which investigated whether a non-clinical community-based sample of males self-reported higher or lower psychopathic traits following stratification into a high performing group (upper 25%; *n* = 250) or low performing group (lower 25%; *n* = 250), on a measure of affective theory of mind. We hypothesised, in line with the literature, that a lower ability to understand other people's mental states would be observed in those who presented with higher ‘levels of psychopathic traits' (Richell et al., 2003; Ali and Chamorro-Premuzic, 2010). A further aim of this study was to investigate the relationship between primary and secondary psychopathic traits and social cognitive outcomes, as well as the relationship between self-reported psychopathy and The Reading the Mind in the Eyes Test Short Form (A and B), in a community sample of males. Our study sample was representative of the community in terms of age (range 18–78), and in terms of normal distribution of outcome scores.

Considering the RMET Short Forms (A and B) from a psychometric perspective, this study reports external validation psychometrics for the RMET-A and RMET-B individually, showing good reliability and an overall medium level of difficulty with a large community-based cohort. The main findings of this study confirm the negative relationship between performance on a measure of social cognition (mentalising) and both primary and secondary psychopathic traits, which was observed for the total RMET test, as well as the individual short forms. Our data is consistent with the literature in showing that decreased performance on measures of social cognition is associated with high self-reported traits of both primary and secondary psychopathy (Richell et al., 2003; Takamatsu and Takai, 2019). To further contextualise this relationship, participants who performed in the upper quartile on the RMET showed no significant relationship to self-report psychopathy scales nor did the middle cohort (*n* = 500). As shown in Figure 1, the interaction effect between the RMET and psychopathic trait subscale trend reported in Table 2, is driven by the performance and self-report of the lower quartile. By recruiting a homogeneous large community-based sample (*N* = 1,000 males), our quartile-based analysis was conduct with robust power and size, with each quartile larger than the total sample of other studies (e.g., Ali and Chamorro-Premuzic, 2010; *N* = 112; 82% Female).

A strength of this study is the large sample, and the inclusion of the RMET Short Forms for validation. A limitation of this study is the reliance on a self-report measure of psychopathy, rather than clinical interview or cohort, though small to moderate associations have been identified between the LSRPS and validated tools e.g., Psychopathy Checklist Revised (Brinkley et al., 2001). A further potential limitation is the specific recruitment of males. However, psychopathy is more frequently seen in males than females and the use of a male-only sample avoided potential floor effects (Lobbestael et al., 2020). Indeed, Muris et al. (2017) suggest that of all Dark Triad traits (narcissism, Machiavellianism, and psychopathy), psychopathy is particularly pronounced in males compared to females. It is acknowledged that these findings cannot be generalised to females. This research further supports the use of the RMET-A and RMET-B in clinical research settings, or with people with high levels of subclinical psychopathic traits.

Our data suggests that there is a significant relationship between social cognitive function and self-reported traits of psychopathy, given the difference in significant associations between the measures depending on whether participants were within the upper or lower quartile for the RMET-36. Due to the cross-sectional survey based nature of this study, a causal hypothesis between poor social cognition and elevated psychopathic traits cannot be examined, though this may be an avenue for future research.

## Data Availability Statement

The raw data supporting the conclusions of this article will be made available by the authors, without undue reservation.

## Ethics Statement

The studies involving human participants were reviewed and approved by The Queens University Belfast Research Ethics Committee (REC REF: EPS 20_32). The patients/participants provided their written informed consent to participate in this study.

## Author Contributions

GC contributed to the data collection. TB contributed to the statistical analysis. All authors contributed to the design and development of the study, as well as writing and reviewing the manuscript.

## Conflict of Interest

The authors declare that the research was conducted in the absence of any commercial or financial relationships that could be construed as a potential conflict of interest.
